# Intractable liver abscess and positive string test: Think *Klebsiella* hypervirulent strain

**DOI:** 10.1016/j.idcr.2022.e01447

**Published:** 2022-02-07

**Authors:** Jeanne Godon, Sylvia Das-Neves, Thomas Blanchot, Lionel Piroth, Mathieu Blot

**Affiliations:** a Department of Infectious Diseases, François Mitterrand University Hospital, Dijon, France; b Laboratory of Bacteriology, François Mitterrand University Hospital, Dijon, France; c CHU Dijon Bourgogne, INSERM, Université de Bourgogne, CIC 1432, Module Épidémiologie Clinique, F21000 Dijon, France; d Lipness Team, INSERM Research Centre LNC-UMR1231 and LabEx LipSTIC, University of Burgundy, Dijon, France

**Keywords:** Computed tomography scan, CT-scan

A 23-year-old Nigerian man with no medical history presented with a 24 hour history of abdominal pain and vomiting. He lived in France without any travels for 3 years. His temperature was 38.5 °C and his abdomen was painful to palpation. Biological screening showed a high level of C-reactive protein (438 mg/L), with leukocytosis (12,000/μL, neutrophils 9000/μL). An abdominal computed tomography scan (CT-scan) revealed a 40 mm liver lesion located in the segment VII (Fig. 1A). Blood cultures were positive for *Klebsiella pneumoniae* without acquired antibacterial resistance. The string test to assess for hypermucoviscosity was positive (Fig. 1B). He was treated with cefotaxime 100 mg/kg/d. However, he experienced persistence of fever, and worsening of his abdominal pain. A new abdominal CT-scan (day 3) showed a progression of a multicompartmental lesion (Fig. 1C). A radio-controlled drainage of the lesion was thus performed.

**Fig. 1:**
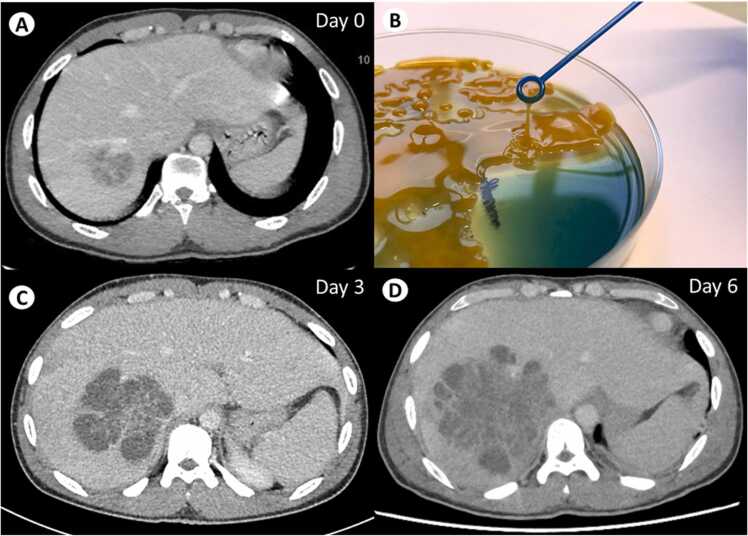
Abdominal computed tomography scan at the admission of the patient (day 0) showing a 40 mm liver abscess (A). Positive string test of the hypermucous *Klebsiella pneumoniae* strain recovered from blood cultures (B). Abdominal computed tomography scan performed at day 3 (C) and 6 (D) following clinical deterioration and showing a progression in size of the multicompartmental liver lesion.

Due to the onset of peritoneal irritation, a new abdominal CT-scan (day 6) was performed and showed a progression of the liver lesion associated with an intraperitoneal fluid effusion (Fig. 1D). He underwent a surgical flattening of the abscess, which was complicated with a hemorrhagic shock requiring vasopressors, transfusion and surgical packing. Intraoperative samples culture grew with the same *Klebsiella pneumoniae*. Whole genome sequencing by illumina revealed a capsular type KL2 with presence of genes associated with hypervirulence, namely roBCDN and iucABCD/iutA. Outcome was then favorable under cefotaxime (total of 37 days) and then ciprofloxacin 500 mg bid for 28 days. This rapidly progressive liver abscess associated with an hypermucous *Klebsiella pneumoniae* phenotype (positive string test) was highly suggestive of a hypervirulent strain. Clinicians should be aware of this picture, especially since hypervirulent *Klebsiella* is now recognized as an emerging pathogen worldwide.

## Contributors

All the authors were involved in the diagnosis and clinical care of the patient, and the writing and approval of the manuscript. Written consent for publication was obtained from the patient.

## Declaration of Competing Interest

None to disclosed.

